# Commentary: Immune cell infiltration and prognostic index in cervical cancer: insights from metabolism-related differential genes

**DOI:** 10.3389/fimmu.2024.1446741

**Published:** 2024-09-19

**Authors:** Fangshi Xu, Jiawei Lai

**Affiliations:** ^1^ Department of Vascular Surgery, The Second Affiliated Hospital of Xi’an Jiaotong University, Xi’an, Shaanxi, China; ^2^ Department of Urology, Shaanxi Provincial People’s Hospital, Xi’an, Shaanxi, China

**Keywords:** bioinformatic technology, machine learning algorithms, prognostic models, cancer, clinical cohort

## Introduction

With the ongoing advancement of bioinformatic technology, utilizing public transcriptome data to predict the clinical outcomes of cancer patients has become standard. A recent study by Ma B et al., titled ‘Immune cell infiltration and prognostic index in cervical cancer: insights from metabolism-related differential genes,’ served as an inspiration for us. This study introduced a novel metabolism-related (MR) model designed to enhance the prognostic and therapeutic evaluations of patients with cervical cancer (CC). The model demonstrated significant prognostic value and was closely linked to the levels of immune cell infiltration and the response to immunotherapy. Although the study was well-designed and analyzed, it warrants further enhancements in the selection of machine learning algorithms, comprehensive bioinformatic evaluations, and validation through extensive clinical cohorts.

## Selection of machine learning algorithms

Currently, lasso regression is the most frequently used machine learning approach for clinical modeling (2). Similarly, the MR model was also reliant on lasso regression for construction (1). In 2024 alone, more than 800 studies have utilized this method to establish clinical assessment models in various cancers. The reasons for its wide application are predominantly due to that lasso regression effectively handles the dilemma of variable selection and model overfitting through creating a penalty function λ (3).

At present, other machine learning algorithms in exception with lasso regression have also been widely applied in the clinical modeling process owing to their particular advantages. For instance, the support vector machine (SVM) is a classical supervised learning algorithm, which has expertise in addressing the issues referring to multi-classification and sample disequilibrium (4). Yu Y et al. have utilized this method to characterize heart failure (5). As for random forest, it has excelled when dealing with non-linear data and complex relational data (6), by which Zhang Y et al. constructed a prediction model for deep vein thrombosis in patients with digestive system tumors (7). Clearly, lasso regression is not the only option in clinical modeling. Notably, the original intention of modeling is to accurately predict survival outcome or therapeutic response of cancer patients, which can be evaluated by C-index or area under curve (AUC) value (8). This elicits a question: Does the models constructed by lasso regression have the best predictive performance, or is lasso regression the optimal solution for modeling?

To address this point, Liu Z et al. demonstrated a remarkable research strategy, termed comparison of multiple machine learning algorithms (9). In their study, 101 kinds of prediction models were fitted based on various machine learning algorithms and their combinations, such as support vector machine-recursive feature elimination (SVM-RFE), random survival forest (RSF), and Ridge method. They then calculated the C-index of each model in all validation cohorts and found the optimal solution for immune lncRNA signature in colorectal cancer (CRC), which was the combination of lasso regression and step cox regression. Therefore, there may be better approaches for fitting the MR model, which awaits verification by comparing multiple machine learning algorithms.

Nonetheless, the advantages of lasso regression over other algorithms should not be overlooked. First, the model fitted by lasso regression offers significant interpretability (10). In contrast, models such as random forest, SVM, XGBoost, and artificial neural network (ANN) often result in ‘black box’ models, which are challenging to interpret internally. This difficulty arises because the predictive results of black box models can typically only be explained by the correlations between input and output, without providing a specific interpreting or reasoning process (11). Furthermore, black box models are often nonlinear, making their decision boundaries more elusive than those of the linear models well-represented by lasso regression. Second, lasso regression is adept at addressing model overfitting and high model complexity. However, other algorithms may not always be competent. For example, when no multicollinearity exists between independent variables, Ridge regression may diminish the predictive performance of the constructed model (12). Similarly, the random forest model is susceptible to overfitting on certain noisy features, and its training time is relatively long due to its dependence on training multiple decision trees simultaneously. Therefore, although lasso regression may not always excel in diagnostic performance, it remains a stable solution with low fault tolerance for most clinical analyses.

## Comprehensive bioinformatics evaluation

Prognostic analysis is a crucial component of personalized cancer medicine and represents a primary concern for patients. In their study, Ma B et al. evaluated the survival differences between patients with high and low MR risks and the accuracy of the MR model in predicting the overall survival rate (OSR) at 1, 3, and 5 years (1). However, other prognostic properties also merit further exploration. For instance, clinical subgroup analyses could test the predictive capacities of the MR model in cancer patients at different clinical stages (13). Additionally, while the AJCC and TNM systems are established foundations for cancer prognostic assessments (14), replacing them with the MR score is impractical. It could be clinically more significant to investigate whether the MR risk score could enhance the predictive accuracy or decision-making benefits of the AJCC or TNM systems using decision curve analysis (DCA) (15).

Regarding immunotherapy response, Ma B et al. explored the associations between MR risk score and Tumor Immune Dysfunction and Exclusion (TIDE) score, tumor mutation burden (TMB), and expressions of immune checkpoints (ICs). These are considered valuable biomarkers for predicting the efficacy of immune checkpoint inhibitors (ICIs) (16–18). Notably, some clinical cohorts could be used for therapeutic effect analysis due to their available transcriptome and clinical data.

Compared to the work of Ma B et al., Betancor YZ et al. demonstrated some advantages in predicting therapeutic response (19). They utilized real-world evidence from three related clinical trials (CheckMate-009, CheckMate-010, and CheckMate-025) to assess the predictive capacity of a three-gene model for anti-PD-1 blockade therapy, highlighting its effectiveness in actual clinical practice. Additionally, considering that nivolumab is the first-line treatment for advanced renal cell carcinoma (RCC), the topic chosen by Betancor YZ et al. may hold more appeal for researchers in this field. Clearly, the research of Ma B et al., particularly in prognostic and immunotherapy analyses, could be further refined with more comprehensive bioinformatic investigations.

## Validation of extensive clinical cohort

Although prediction models are increasingly prevalent, only a minority have undergone validation using the authors’ own clinical cohorts, significantly limiting their clinical applicability (20). Regrettably, the prognostic value of the MR model was validated only in an external cohort (GSE52904 dataset) and not in more comprehensive clinical cohorts.

There are two primary drawbacks to relying solely on public data. First, demographic differences among various clinical cohorts can introduce selection bias. For example, the age range of patients in the GSE52904 dataset spanned from 24 to 74 years (Mean=50.5 years) (21), whereas the average age in another cervical cancer cohort, GSE6791, was 43.9 years (22). Additionally, there were discrepancies among patients in terms of race, clinical stages, pathological types, and follow-up times across different cohorts. Thus, using external clinical data could help minimize this selection bias to some extent. Second, the clinical validation within their own centers is a critical first step toward the clinical application of constructed models. If models developed by authors do not perform well in their own clinical cohorts, it is challenging to convince surgeons of their clinical potential and value.

Moreover, since the modeling process tends towards purely mathematical operations, the lack of clinical data correction can cause the constructed model to deviate from actual circumstances. For instance, a six-genes ZNF family model developed for clinical assessments of esophageal cancer (ESCS) (23) showed no significant differences in ZNF502 expressions between tumor and normal clinical samples, as determined by PCR. This undoubtedly diminishes the credibility of this model to some extent. Collectively, it is crucial to advocate for more extensive clinical validation.

## Some suggestions on clinical data validation

Validating models or risk signatures on external clinical cohorts, such as those from a researcher’s own center, significantly enhances their credibility, although this path is challenging yet worthwhile. From our perspective, there are two critical enabling factors. First, long-term and meticulous follow-up is essential. Prognostic assessment plays a pivotal role in individualized cancer therapy. Collecting detailed data on patient survival or therapeutic responses will greatly inform these issues. Second, the construction of a genome-wide library is crucial. With the rapid development of genomics, selecting appropriate patients and performing exon sequencing on their clinical samples can link molecular features to clinical phenotypes (24), enhancing our understanding of disease pathogenesis. However, these strategies are accompanied by considerable human and economic costs.

## Conclusions

In this manuscript, we propose several approaches to refine the MR risk score, thereby enhancing its clinical application. First, optimizing the machine learning algorithm may improve its prediction performance. Second, the increased use of bioinformatics technologies, such as Decision Curve Analysis (DCA), is instrumental in assessing the clinical value of the MR risk score. Third, further validation using external clinical data will enhance the credibility of the MR risk score.

The rapid expansion of bioinformatics technology has broadened the scope of cancer research. However, the majority of bioinformatics research encounters common issues, such as the selection of modeling algorithms and the scarcity of real-world data. These limitations significantly restrict the clinical application of novel prediction models. We highlight three improvement measures to address these deficiencies, which will contribute to a better understanding of the application of bioinformatics in the cancer field.
